# Comparing the Efficacy of Large Language Models ChatGPT, BARD, and Bing AI in Providing Information on Rhinoplasty: An Observational Study

**DOI:** 10.1093/asjof/ojad084

**Published:** 2023-09-14

**Authors:** Ishith Seth, Bryan Lim, Yi Xie, Jevan Cevik, Warren M Rozen, Richard J Ross, Mathew Lee

## Abstract

**Background:**

Large language models (LLMs) are emerging artificial intelligence (AI) technologies refining research and healthcare. However, the impact of these models on presurgical planning and education remains under-explored.

**Objectives:**

This study aims to assess 3 prominent LLMs—Google's AI BARD (Mountain View, CA), Bing AI (Microsoft, Redmond, WA), and ChatGPT-3.5 (Open AI, San Francisco, CA) in providing safe medical information for rhinoplasty.

**Methods:**

Six questions regarding rhinoplasty were prompted to ChatGPT, BARD, and Bing AI. A Likert scale was used to evaluate these responses by a panel of Specialist Plastic and Reconstructive Surgeons with extensive experience in rhinoplasty. To measure reliability, the Flesch Reading Ease Score, the Flesch–Kincaid Grade Level, and the Coleman–Liau Index were used. The modified DISCERN score was chosen as the criterion for assessing suitability and reliability. A *t* test was performed to calculate the difference between the LLMs, and a double-sided *P*-value <.05 was considered statistically significant.

**Results:**

In terms of reliability, BARD and ChatGPT demonstrated a significantly (*P* < .05) greater Flesch Reading Ease Score of 47.47 (±15.32) and 37.68 (±12.96), Flesch–Kincaid Grade Level of 9.7 (±3.12) and 10.15 (±1.84), and a Coleman–Liau Index of 10.83 (±2.14) and 12.17 (±1.17) than Bing AI. In terms of suitability, BARD (46.3 ± 2.8) demonstrated a significantly greater DISCERN score than ChatGPT and Bing AI. In terms of Likert score, ChatGPT and BARD demonstrated similar scores and were greater than Bing AI.

**Conclusions:**

BARD delivered the most succinct and comprehensible information, followed by ChatGPT and Bing AI. Although these models demonstrate potential, challenges regarding their depth and specificity remain. Therefore, future research should aim to augment LLM performance through the integration of specialized databases and expert knowledge, while also refining their algorithms.

**Level of Evidence: 5:**

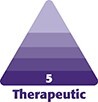

Rhinoplasty remains one of the most sought-after surgical procedures across the globe.^1^ In 2018, the United States saw over 200,000 such operations, making it the third most common plastic surgery performed in the nation.^2^ The nose is the central focal point of the face, with its proportions and symmetry having a significant impact on overall facial aesthetics.^1^

With the recent advancements in artificial intelligence (AI) and natural language processing, large language models (LLMs) have demonstrated remarkable capabilities in the surgical field.^3-7^ The transformative power of AI has pervaded a plethora of domains, including healthcare, where it has revolutionized diagnostics, treatment planning, and patient care.^8^ In recent times, AI-driven LLMs, such as ChatGPT (Open AI, San Francisco, CA), BARD (Google, Mountain View, CA), and Bing AI (Microsoft, Redmond, WA), have gained considerable attention for their ability to comprehend and generate human-like text based on massive amounts of training data.^4,9,10^ The integration of AI techniques holds tremendous potential to reshape the field of plastic surgery by providing insights into patient-specific rhinoplasty outcomes, surgical planning, and postoperative care. Moreover, the utilization of LLMs can facilitate efficient extraction and analysis of scientific literature, enhancing the understanding of best practices and potential complications associated with rhinoplasty.^11^ However, alongside the potential benefits, it is imperative to address the challenges and limitations associated with deploying AI-driven language models in a medical context, to ensure the reliability, accuracy, and ethical use of generated information.

This study aims to investigate the efficacy of employing LLMs in obtaining and synthesizing information about rhinoplasty. We assess the mechanisms driving these models to appraise their capacity to augment presurgical planning or medical decision making. Additionally, we scrutinize the ethical and pragmatic aspects of incorporating AI-powered language models into clinical settings, highlighting potential advantages and challenges.

## METHODS

We engaged ChatGPT-3.5, BARD, and Bing AI with a series of 6 questions targeting various technical aspects of rhinoplasty. These queries were devised by 3 board-certified plastic surgeons who were fellows of the Royal Australasian College of Surgeons with over 25 years of combined experience performing Rhinoplasty and expertise in facial reconstructive surgery. The aim was to evaluate the breadth and depth of the LLMs’ knowledge of rhinoplasty. The accuracy of responses was determined by comparing them with current healthcare guidelines for rhinoplasty and through evaluation by the panel of plastic surgeons through a Likert scale (Table 1). If any differences in the Likert scale arose, these were discussed until consensus was achieved. During the process, the LLMs were requested to supply 5 high-quality references to substantiate their responses. The references and literature were then assessed for relevance and quality and compared to our preliminary database searches on PubMed (National Institutes of Health, Bethesda, MD), Web of Science (Clarivate Analytics, London, UK), Scopus (Elsevier, Amsterdam, the Netherlands), EMBASE (Elsevier), Cochrane CENTRAL (Wiley, Hoboken, NJ), and Google search. Lastly, the LLM responses were quantitatively analyzed using validated tools (Table 2). The LLMs were requested to limit their answers to 200 words. To standardize response comparisons and mimic real-world preference for brevity, this has been validated in previous LLM studies.^7,12^ To measure the reliability of the response, the Flesch Reading Ease Score, the Flesch–Kincaid Grade Level, and the Coleman–Liau Index were utilized. The Flesch Reading Ease Score, on a scale of 0 to 100, indicates the readability of the text—a higher score translates to easier comprehension. The Flesch–Kincaid Grade Level and the Coleman–Liau Index, with scales ranging from 0 to ∞, reflect the complexity of the text and the education level required for understanding it, respectively, and in both instances, a higher score implies more complexity. We also employed the modified DISCERN score, which ranges from 16 to 80. A higher score on this scale represents superior quality and more balanced treatment option information. This score was used to assess the appropriateness of the response, thereby ensuring the provided information's quality and relevance for patients. *T* test was employed to compare the differences between the 3 LLMs, and a *P*-value <.05 was considered statistically significant.

**Table 1.** Evaluation of the Reponses of Large Language Model Platforms

Criteria	ChatGPT (Open AI)	Bing AI (Microsoft)	Google's BARD
The large language model provides accurate answers to questions.	[ ] 1—Strongly disagree [ ] 2—Disagree [ ] 3—Neither agree or disagree [x] 4—Agree [ ] 5—Strongly agree	[ ] 1—Strongly disagree [ ] 2—Disagree [x] 3—Neither agree or disagree [ ] 4—Agree [ ] 5—Strongly agree	[ ] 1—Strongly disagree [ ] 2—Disagree [ ] 3—Neither agree or disagree [x] 4—Agree [ ] 5—Strongly agree
The large language model is reliable when generating factual information.	[ ] 1—Strongly disagree [ ] 2—Disagree [ ] 3—Neither agree or disagree [x] 4—Agree [ ] 5—Strongly agree	[ ] 1—Strongly disagree [ ] 2—Disagree [x] 3—Neither agree or disagree [ ] 4—Agree [ ] 5—Strongly agree	[ ] 1—Strongly disagree [ ] 2—Disagree [ ] 3—Neither agree or disagree [x] 4—Agree [ ] 5—Strongly agree
The large language model is proficient at understanding complex questions and providing appropriate answers.	[ ] 1—Strongly disagree [ ] 2—Disagree [ ] 3—Neither agree or disagree [x] 4—Agree [ ] 5—Strongly agree	[x] 1—Strongly disagree [ ] 2—Disagree [ ] 3—Neither agree or disagree [ ] 4—Agree [ ] 5—Strongly agree	[ ] 1—Strongly disagree [x] 2—Disagree [ ] 3—Neither agree or disagree [ ] 4—Agree [ ] 5—Strongly agree
The large language model provides comprehensive information when answering questions.	[ ] 1—Strongly disagree [ ] 2—Disagree [ ] 3—Neither agree or disagree [x] 4—Agree [ ] 5—Strongly agree	[ ] 1—Strongly disagree [x] 2—Disagree [ ] 3—Neither agree or disagree [ ] 4—Agree [ ] 5—Strongly agree	[ ] 1—Strongly disagree [ ] 2—Disagree [ ] 3—Neither agree or disagree [x] 4—Agree [ ] 5—Strongly agree
The large language model generates content that covers all relevant aspects of a subject.	[ ] 1—Strongly disagree [ ] 2—Disagree [ ] 3—Neither agree or disagree [x] 4—Agree [ ] 5—Strongly agree	[ ] 1—Strongly disagree [x] 2—Disagree [ ] 3—Neither agree or disagree [ ] 4—Agree [ ] 5—Strongly agree	[ ] 1—Strongly disagree [ ] 2—Disagree [x] 3—Neither agree or disagree [ ] 4—Agree [ ] 5—Strongly agree
The large language model is able to provide in-depth information for a wide range of topics.	[ ] 1—Strongly disagree [ ] 2—Disagree [ ] 3—Neither agree or disagree [x] 4—Agree [ ] 5—Strongly agree	[ ] 1—Strongly disagree [x] 2—Disagree [ ] 3—Neither agree or disagree [ ] 4—Agree [ ] 5—Strongly agree	[ ] 1—Strongly disagree [ ] 2—Disagree [x] 3—Neither agree or disagree [ ] 4—Agree [ ] 5—Strongly agree
The large language model is a valuable source of general knowledge.	[ ] 1—Strongly disagree [ ] 2—Disagree [ ] 3—Neither agree or disagree [ ] 4—Agree [x] 5—Strongly agree	[ ] 1—Strongly disagree [x] 2—Disagree [ ] 3—Neither agree or disagree [ ] 4—Agree [ ] 5—Strongly agree	[ ] 1—Strongly disagree [ ] 2—Disagree [ ] 3—Neither agree or disagree [x] 4—Agree [ ] 5—Strongly agree
The large language model is well-versed in a variety of subjects.	[ ] 1—Strongly disagree [ ] 2—Disagree [ ] 3—Neither agree or disagree [ ] 4—Agree [x] 5—Strongly agree	[ ] 1—Strongly disagree [x] 2—Disagree [ ] 3—Neither agree or disagree [ ] 4—Agree [ ] 5—Strongly agree	[ ] 1—Strongly disagree [ ] 2—Disagree [ ] 3—Neither agree or disagree [x] 4—Agree [ ] 5—Strongly agree
The large language model can provide useful insights and perspectives on various topics.	[ ] 1—Strongly disagree [ ] 2—Disagree [ ] 3—Neither agree or disagree [x] 4—Agree [ ] 5—Strongly agree	[ ] 1—Strongly disagree [x] 2—Disagree [ ] 3—Neither agree or disagree [ ] 4—Agree [ ] 5—Strongly agree	[ ] 1—Strongly disagree [ ] 2—Disagree [x] 3—Neither agree or disagree [ ] 4—Agree [ ] 5—Strongly agree
The large language model rarely makes errors when referencing sources.	[ ] 1—Strongly disagree [x] 2—Disagree [ ] 3—Neither agree or disagree [ ] 4—Agree [ ] 5—Strongly agree	[ ] 1—Strongly disagree [ ] 2—Disagree [x] 3—Neither agree or disagree [ ] 4—Agree [ ] 5—Strongly agree	[ ] 1—Strongly disagree [x] 2—Disagree [ ] 3—Neither agree or disagree [ ] 4—Agree [ ] 5—Strongly agree
The large language model is consistent in providing accurate citations.	[ ] 1—Strongly disagree [x] 2—Disagree [ ] 3—Neither agree or disagree [ ] 4—Agree [ ] 5—Strongly agree	[ ] 1—Strongly disagree [ ] 2—Disagree [x] 3—Neither agree or disagree [ ] 4—Agree [ ] 5—Strongly agree	[ ] 1—Strongly disagree [ ] 2—Disagree [x] 3—Neither agree or disagree [ ] 4—Agree [ ] 5—Strongly agree

**Table 2.** Readability and Reliability of the Responses of Large Language Models

Model	Prompts	Readability			Suitability
		Flesch reading ease score	Flesch–Kincaid grade level	Coleman–Liau index	DISCERN score
ChatGPT (Open AI)	Correcting internal valve dysfunction	42.98	9.56	15	42
	Correcting external valve collapse	41.43	9.69	16	42
	Correcting caudal septal dislocation	33.97	10.59	6	44
	Managing turbinate hypertrophy	13.27	13.62	12	41
	Managing tip support after submucous resection	49.02	8.46	18	43
	When to do nasal bone fractures in rhinoplasty	45.42	8.99	12.17	41
Mean (SD)		37.68 (±12.96)	10.15 (±1.84)	12.00 (5.10)	42.17 (±1.17)
Google's BARD	Correcting internal valve dysfunction	61.26	6.98	10	44
	Correcting external valve collapse	61.63	7.25	9	47
	Correcting caudal septal dislocation	36.08	11.27	13	42
	Managing turbinate hypertrophy	23.03	15.2	14	48
	Managing tip support after submucous resection	54.89	8.15	9	47
	When to do nasal bone fractures in rhinoplasty	47.91	9.4	10	50
Mean (SD)		47.47 (±15.32)	9.71 (±3.12)	10.83 (±2.14)	46.33 (±2.88)
Bing AI(Microsoft)	Correcting internal valve dysfunction	25.95	20	16	39
	Correcting external valve collapse	10.17	20.86	14	30
	Correcting caudal septal dislocation	14.41	19.2	14	36
	Managing turbinate hypertrophy	4.89	18.37	19	38
	Managing tip support after submucous resection	NA	25.34	14	NA
	When to do nasal bone fractures in rhinoplasty	36.03	13.56	12	46
Mean (SD)		18.29 (±12.59)	18.25 (±4.84)	12.00 (5.10)	35.75 (±4.03)

NA, not applicable; SD, standard deviation.

The inclusion criteria for this study encompassed the initial response given by the LLMs to each question. No exclusion criteria were applied to the answers generated. Since this study entailed an observational case study of a publicly available AI LLM, no institutional ethical approval was required.

## RESULTS

In response to the first inquiry in the Figure, ChatGPT accurately delineated surgical interventions for internal nasal valve dysfunction, emphasizing the importance of enhancing cross-sectional area but overlooked discussing the angle between upper lateral cartilages and the anterior septal edge. Among 5 referenced sources, only 2 were found in the literature,^13,14^ 1 being contextually relevant.^13^ BARD provided a contextual overview, briefly discussing the mechanism and potential side effects, before identifying spreader, Alar batten, and Butterfly grafts,^15,16^ but failed to discuss the spreader graft's limitations in expanding a collapsed lateral nasal wall as noted by Teymoortash et al. Only 1 of its 5 references could be corroborated in the literature. Bing AI listed the same techniques as BARD, providing a brief historical context for spreader grafts and the primary etiology of internal nasal valve dysfunction. It produced legitimate references, yet only 2 were academic. Assessing the responses, ChatGPT presented a broader range of surgical options, including flaring sutures, as a potential alternative to grafts.

**Figure.**
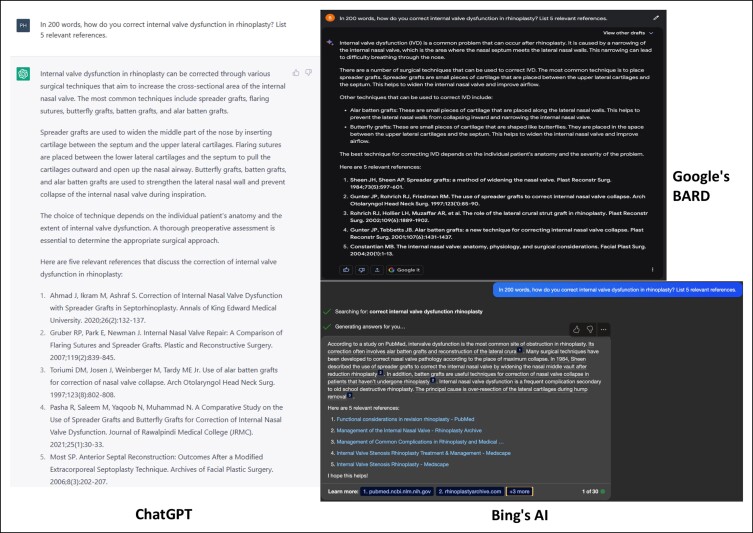
In 200 words, how do you correct internal valve dysfunction in rhinoplasty? List 5 relevant references.

ChatGPT's response to the second inquiry in Supplemental Figure 1 followed a similar pattern as the first. It discussed fortifying the lateral nasal wall but ignored the significance of the lateral crus and nasal septum in external nasal valve function. It correctly listed methods for nasal tip deprojection, alar rim reinforcement, and crura realignment,^17^ although its 5 references proved to be false. BARD accurately outlined external valve collapse, its dysfunction, and its causes, followed by a brief discussion of 3 surgical interventions. Like ChatGPT, all its references were erroneous. Bing AI's shorter response focused on the general concept of deprojecting the over-projected nose, but only hinted at the use of lateral crura strut and alar grafts, failing to expand further. None of its references were academic in nature. Overall, ChatGPT provided the most detailed and diverse surgical options for the correction of external valve collapse.

Supplemental Figure 2 shows the LLMs’ management for caudal septal dislocation. ChatGPT accurately mentioned the tongue-in-groove technique,^18^ but misattributed techniques such as medial crural overlay, caudal septal extension, and transdermal suture to septal dislocation correction, which are typically adjuncts for nasal tip projection correction, and not primary methods for caudal septal dislocation. Only 1 of its references could be found in the literature, but it related to nasal tip contouring,^19^ not supporting ChatGPT's response. BARD focused on surgical techniques, but used vague terminologies such as “Suturing,” “Grafting,” and “Septoplasty,” All its references were incorrectly cited, missing author names, journal volumes, and page numbers. Bing AI was again brief, mainly discussing septoplasty and vaguely discussing open approaches and grafts. It was able to provide 1 academic article, matching ChatGPT in this response. Consequently, ChatGPT outperformed both by offering more detailed and numerous surgical options.

The response provided by ChatGPT to the fourth prompt in Supplemental Figure 3 exhibited comparable structure to its previous responses. It appropriately emphasized reducing tissue size to improve nasal airflow but overlooked the potential impacts on mucociliary clearance and local immune function. It provided a list of surgical techniques for treating this condition,^20,21^ although its references were again unverifiable. BARD underscored the pathophysiology and symptoms of turbinate hypertrophy and discussed 2 surgical corrections. Analogous to its previous response, it omitted crucial citation details like author names and journal volumes, while providing a link to a specialist’s website, resulting in low-quality references. Bing AI offered a larger array of options, including nonsurgical ones such as nasal sprays and antihistamines, but only suggested inferior turbinate resection as a surgical option.^22^ It marginally outperformed ChatGPT and BARD in this instance by supplying 2 academic articles.

In ChatGPT's response to the fifth prompt in Supplemental Figure 4. The analysis exhibited a relatively restricted purview. It covered nasal tip support in rhinoplasty but overlooked the complex relationship between the junction of the medial and the lateral crurae of each lower lateral cartilage and its effect on tip support.^23,24^ Unfortunately, ChatGPT's references were spurious and could not be verified. BARD provided a brief background on submucous resection's impact on tip support and discussed 3 corrective surgical methods. All of its references were absent in the literature. Bing AI failed to provide any relevant information or recommendations.

The objective of the sixth prompt in Supplemental Figure 5 was to assess ChatGPT's recommendations regarding the timing of osteotomy performance during rhinoplasty. While the response accurately identified 1 rationale for conducting an osteotomy during rhinoplasty, it omitted others, such as addressing open roof deformities.^25^ Although the open and closed approaches were discussed, the response failed to delineate the specific circumstances warranting their application, instead focusing on procedural technicalities. All 5 references provided were truncated and, as such, could not be further analyzed. Regrettably, both BARD and Bing AI erroneously interpreted the question, proposing surgical interventions for fractured nasal bones.

When comparing the 3 LLMs for readability and reliability, BARD consistently outperformed, registering the highest Flesch Reading Ease Score (47.5 ± 15.32), Flesch–Kincaid Grade Level (9.71 ± 3.12), and DISCERN score (46.3 ± 2.88), indicative of superior comprehensibility and medical advice alignment with clinical guidelines. This was followed by ChatGPT, and then Bing AI, which lagged in all categories. The only comparisons that yielded statistically nonsignificant outcomes (*P* > .05) were those between ChatGPT and BARD regarding their readability indices and between ChatGPT and Bing AI when assessing the DISCERN scores. All other comparative analyses demonstrated statistical significance (*P* < .05).

## DISCUSSION

LLMs have ascended to prominence in the medical sector owing to their proficient rapid information retrieval and algorithmic decision-making capabilities.^26^ The integration of AI into the planning of rhinoplasty operations has seen remarkable growth,^27,28^ particularly in the context of the burgeoning utilization of LLMs in healthcare. Given this trajectory, this research study comparatively appraises the potential of Google's BARD, Bing AI, and ChatGPT in enhancing preoperative planning and decision making.

It is worth noting that the LLMs were not intended to serve as a literature search or reference engine. ChatGPT and BARD, machine-learning models trained on diverse internet data, generate text based on statistical models, covering a wide array of topics. However, they cannot source literature to support their content, despite their predictive text generation creating an illusion of authenticity as observed by Saleem et al.^29^ Bing AI, benefiting from real-time internet access, does not share this limitation, but its references are subpar, offering few academic articles, and failing to cite them appropriately. Accordingly, authoring scientific literature still necessitates the guidance and supervision of experts.^30^

ChatGPT demonstrates promise in enhancing preoperative planning in rhinoplasty procedures. Open AI is currently working on a feature that enables ChatGPT to analyze and interpret images, which, when combined with machine-learning algorithms and perioperative photographic patient records, could yield highly accurate predictions of rhinoplasty outcomes. Google also recently announced a forthcoming update to BARD, incorporating image interpretation, including those of medical relevance. This enhancement has significant potential to bolster the presurgical planning phase for rhinoplasties. Such predictions could provide surgeons with innovative ways to plan procedures and identify potential complications.^31^

However, certain limitations within its current algorithm need to be addressed. As shown in the Figure and Supplemental Figures 1 to 5, all 3 LLMs generated potentially incomplete and unreliable lists of surgical management, omitting feasible surgical interventions. Moreover, in Supplemental Figure 5, BARD and Bing AI deviated from the prompt, dedicating much of their response to explaining surgical procedures rather than concentrating on when they should be performed. Additionally, as evidenced in Supplemental Figure 2, certain surgical techniques were inappropriately suggested by ChatGPT for the given scenario. One possible approach to addressing these limitations is refining the prompt, as highlighted by Li et al, who emphasized the importance of properly crafting prompts.^32^ Conversely, before integrating LLMs into medical practice, it is crucial to rectify these algorithmic shortcomings to ensure reliable and accurate information.

BARD demonstrated superior readability and reliability than ChatGPT and Bing AI, making it ideal for nonmedical users and as a preliminary medical advisory tool in medically underserved areas. ChatGPT's responses, although understandable, were less comprehensive partly due to its failure to contextualize the defect and emphasize the need for repair as BARD does. This is crucial as patients may retrieve knowledge from these LLMs and other sources, empowering them to be actively involved in presurgery decision making and planning. Despite Bing AI's complex medical language, it often fell short of a detailed explanation. The study by Zhu et al supports the impact of different training data and preprocessing methods on LLMs’ readability,^33^ suggesting further research could optimize algorithms and improve comprehensibility. Furthermore, the *t* test demonstrated that most comparisons were statistically significant. However, the comparisons pertaining to readability between ChatGPT and BARD, as well as the DISCERN score between ChatGPT and Bing AI, were statistically insignificant (*P* > .05). This implies that ChatGPT may not necessarily be less readable than BARD and more reliable than Bing AI. Hence, the authors agree with the current literature and suggest further studies comparing these LLMs on these parameters to obtain more reliable and statistically significant results.

Concerningly, none of the models assessed the benefits, risks, and postoperative quality of life related to the suggested procedures, which are vital for presurgical planning. Rhinoplasties, for example, can significantly impact nasal functionality and a patient's psychological state postsurgery.^34^ It would have been beneficial if the models had briefly touched on measures such as the Health Measurement Questionnaire and Glasgow Benefit Inventory.^35^ The authors recommend fostering collaborations between AI developers and clinical experts to improve these LLMs’ performance. Using specialized databases and expert knowledge could enhance the models’ accuracy and depth of information. Moreover, ensuring the traceability and credibility of AI-generated content is key to building users’ trust and accountability.

ChatGPT, Google's BARD, and Bing AI are revolutionizing the field with their advanced contextual understanding and predictive capabilities.^36^ Integrated into various applications, they show promise in enhancing preoperative planning, decision making, and patient education. By synthesizing evidence-based recommendations, they exhibit the potential to equip surgeons with the latest best practices. Through the fusion of natural language processing and computer vision, these models may provide insights into patient-specific surgical planning, possibly enhancing precision, safety, and patient outcomes.^37^

Assessing the LLMs’ recommendations for managing various technical aspects of rhinoplasty proved interesting. Multiple factors influence a plastic surgeon's approach to nasal reconstruction, wherein applying inappropriate surgical methods can adversely affect patient outcomes. Consequently, AI tools that provide erroneous recommendations may have legal and ethical ramifications that could implicate both the treating team and the software developer.^38^ Considering the limitations uncovered in this study, all 3 LLMs necessitate significant enhancements before their practical application in rhinoplasty management. Therefore, users should consult experienced plastic surgeons in addition to the 3 models’ recommendations.

In summary, the LLMs demonstrated a moderate grasp of the rhinoplasty-related questions posed, providing logical and easily understandable answers. As LLMs, their primary function revolves around predicting the likelihood of a word sequence based on the context provided by preceding words. While this ability has allowed the LLMs to achieve impressive feats, it also limits their capacity to offer in-depth information. Consequently, this study underscores the limitations of LLMs in delivering detailed knowledge on specialized surgical subjects. Moving forward, the creation of a “scholar”-type LLM tailored for physicians and similar professionals would be valuable. Google Scholar, PubMed, and other databases curate peer-reviewed academic literature, which is highly useful but not directly actionable in a clinical setting. A specialized LLM could potentially fill this gap by distilling complex, peer-reviewed medical information into actionable information for healthcare providers. We anticipate that many of the current limitations will be resolved in time given the rapidly changing nature of this technology, yet, at this point of time, further refinement is necessary for these models to be effectively integrated into clinical practice.

## CONCLUSIONS

Overall, this study demonstrates that using LLMs such as ChatGPT, BARD, and Bing AI for acquiring detailed information on specialized surgical procedures like rhinoplasty has inherent limitations. While these models can offer pertinent and accessible information, inconsistencies and superficial content may be present. It is imperative to scrupulously evaluate the information provided by these AI systems and corroborate it with evidence-based sources and expert insights to ensure accuracy and reliability in rhinoplasty presurgical planning, decision making, and patient education.

## Supplemental Material

This article contains supplemental material located online at www.asjopenforum.com.

## Supplementary Material

ojad084_Supplementary_Data
