# Supplementary material for: Comparing the Efficacy of Large Language Models ChatGPT, BARD, and Bing AI in Providing Information on Rhinoplasty: An Observational Study
Source: Aesthet Surg J Open Forum. 2023 Sep 14;5:ojad084. doi: 10.1093/asjof/ojad084 (PMC10547367; doi:10.1093/asjof/ojad084)
Supplement: ojad084_Supplementary_Data [file ojad084_supplementary_data.zip › 23-0083_Supplemental Figure Legends.docx]

**Supplemental Figure Legends**

**Supplementary Figure 1.** In 200 words, how do you correct external valve collapse in rhinoplasty? List 5 relevant references.

**Supplementary Figure 2.** In 200 words, how do you correct caudal septal dislocation in rhinoplasty? List 5 relevant references.

**Supplementary Figure 3.** In 200 words, how do you manage turbinate hypertrophy in rhinoplasty? List 5 relevant references.

**Supplementary Figure 4.** In 200 words, how do you manage tip support after submucous resection? List 5 relevant references.

**Supplementary Figure 5.** In 200 words, when should you do nasal bone fractures in rhinoplasty? List 5 relevant references.
