# Supplementary figures and images for: Comparing the Efficacy of Large Language Models ChatGPT, BARD, and Bing AI in Providing Information on Rhinoplasty: An Observational Study
Source: Aesthet Surg J Open Forum. 2023 Sep 14;5:ojad084. doi: 10.1093/asjof/ojad084 (PMC10547367; doi:10.1093/asjof/ojad084)

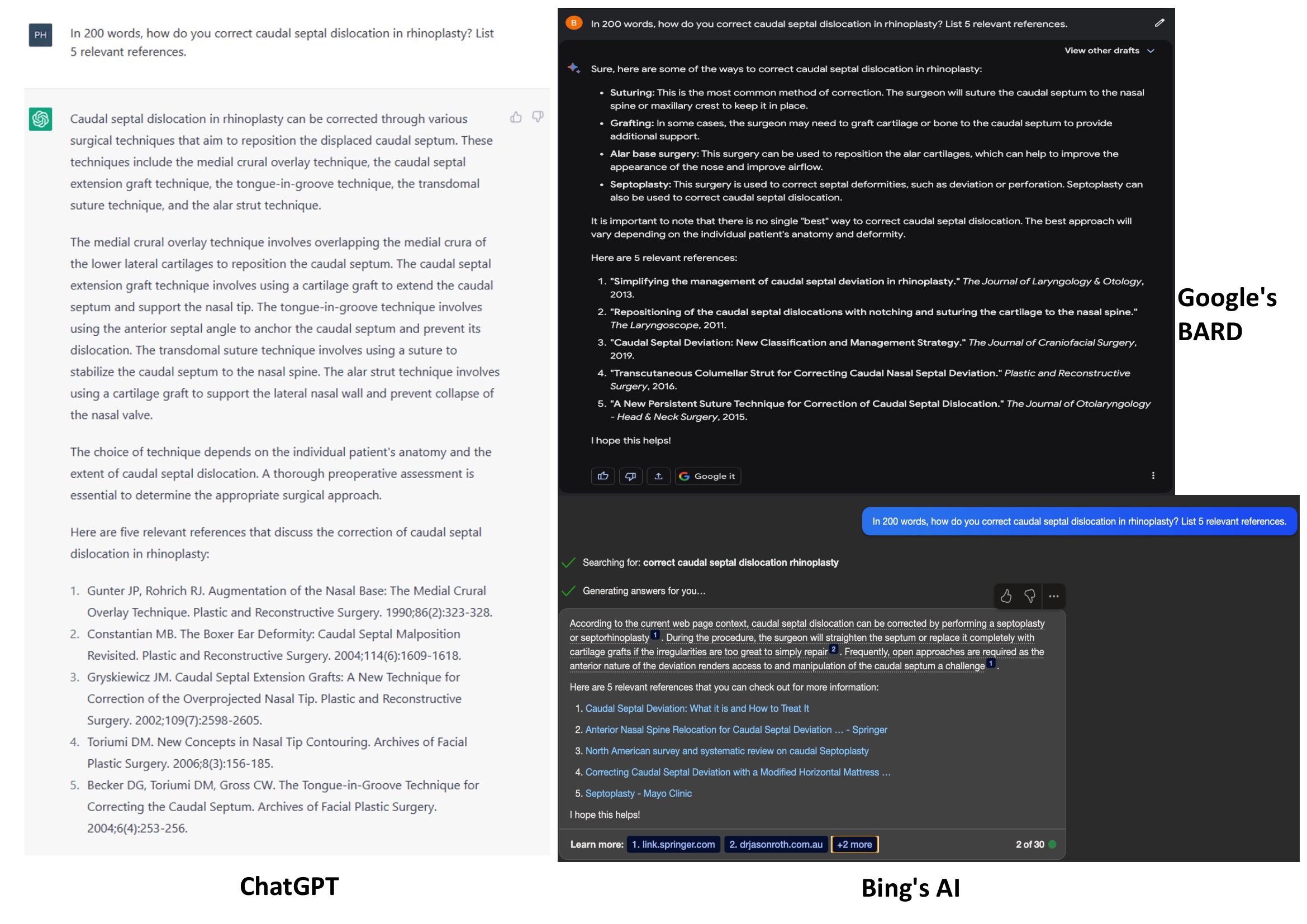

Supplement: ojad084_Supplementary_Data [file ojad084_supplementary_data.zip › Supplementary_Figure_2.tiff]

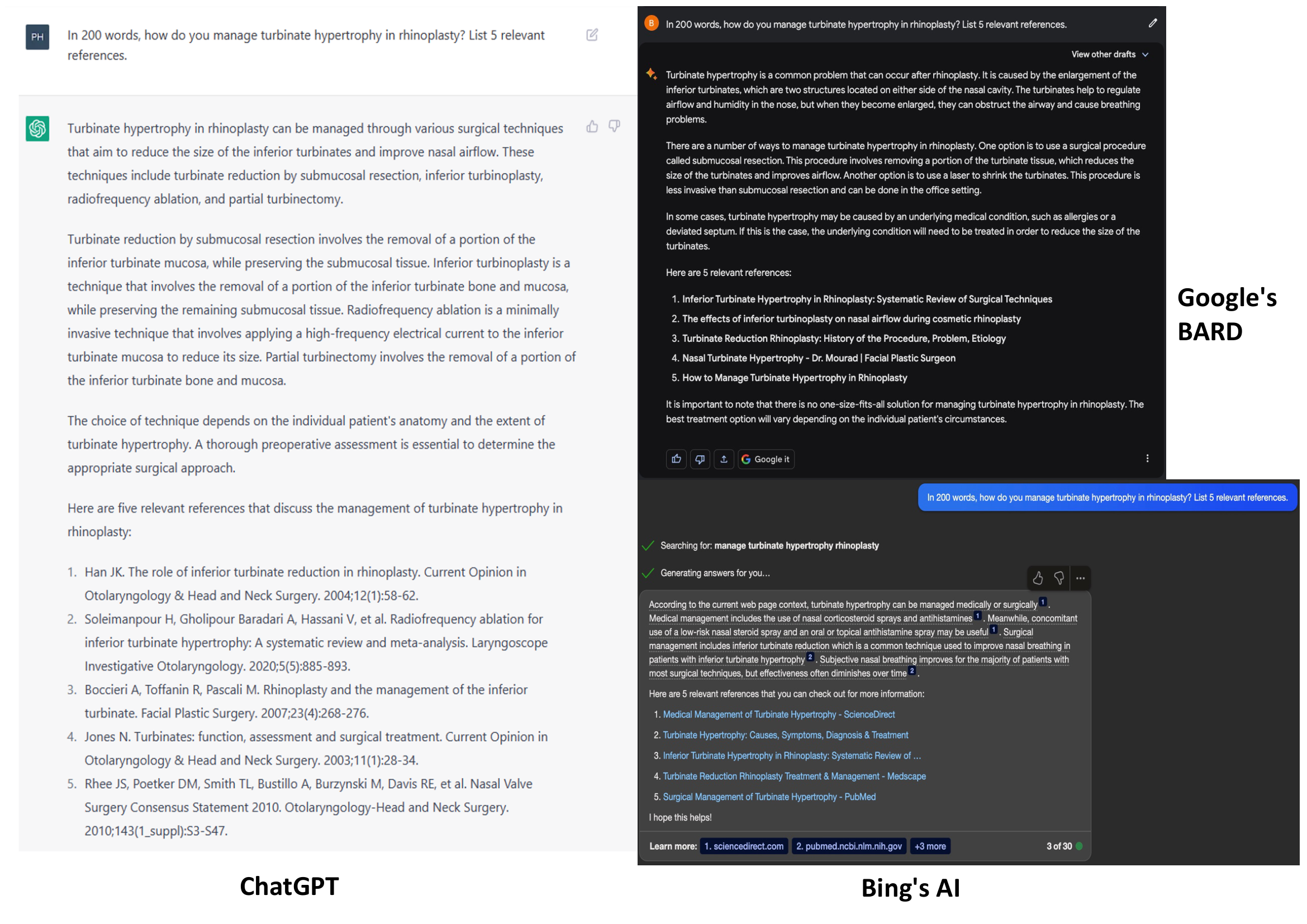

Supplement: ojad084_Supplementary_Data [file ojad084_supplementary_data.zip › Supplementary_Figure_3.tiff]

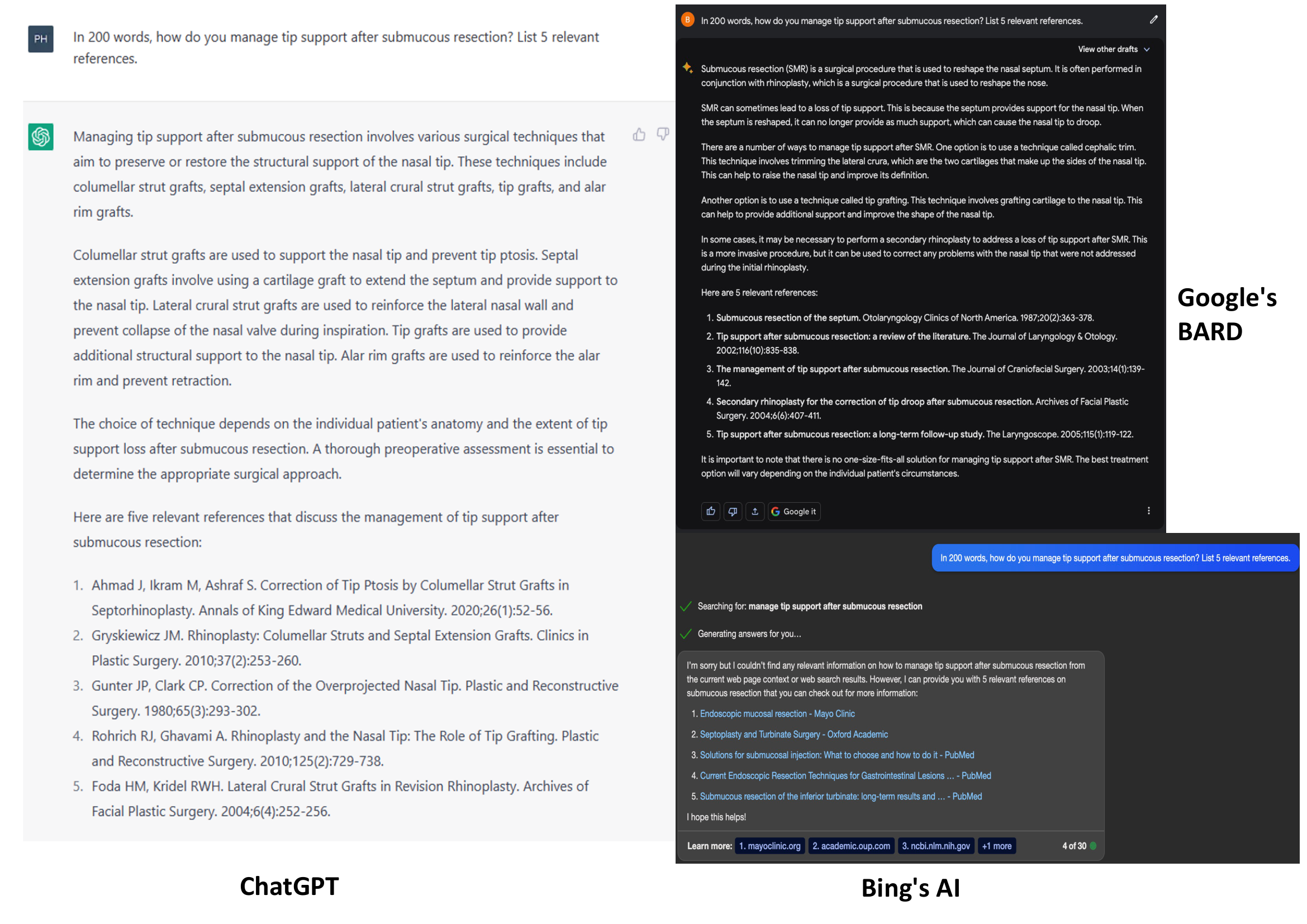

Supplement: ojad084_Supplementary_Data [file ojad084_supplementary_data.zip › Supplementary_Figure_4.tiff]

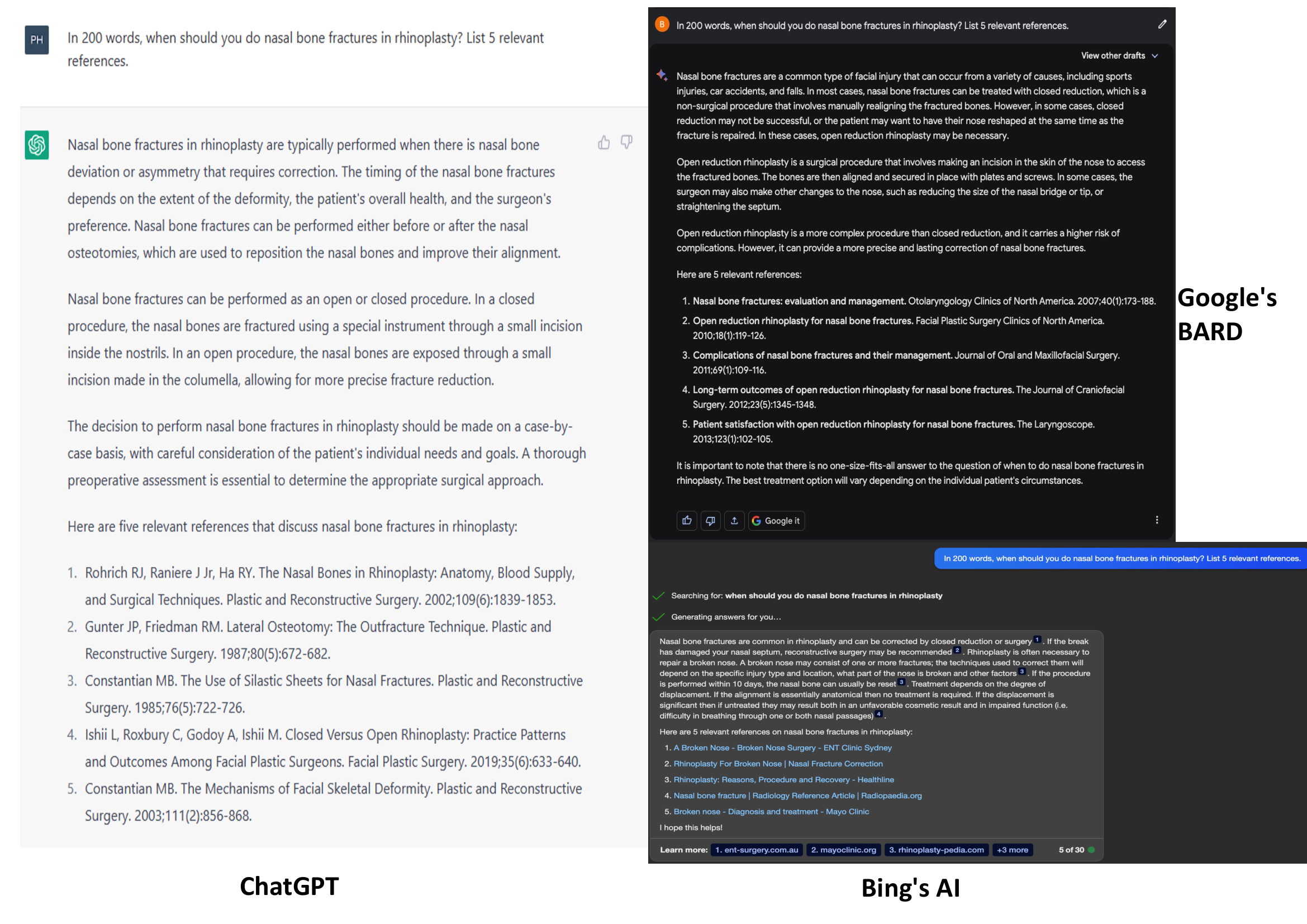

Supplement: ojad084_Supplementary_Data [file ojad084_supplementary_data.zip › Supplementary_Figure_5.tiff]

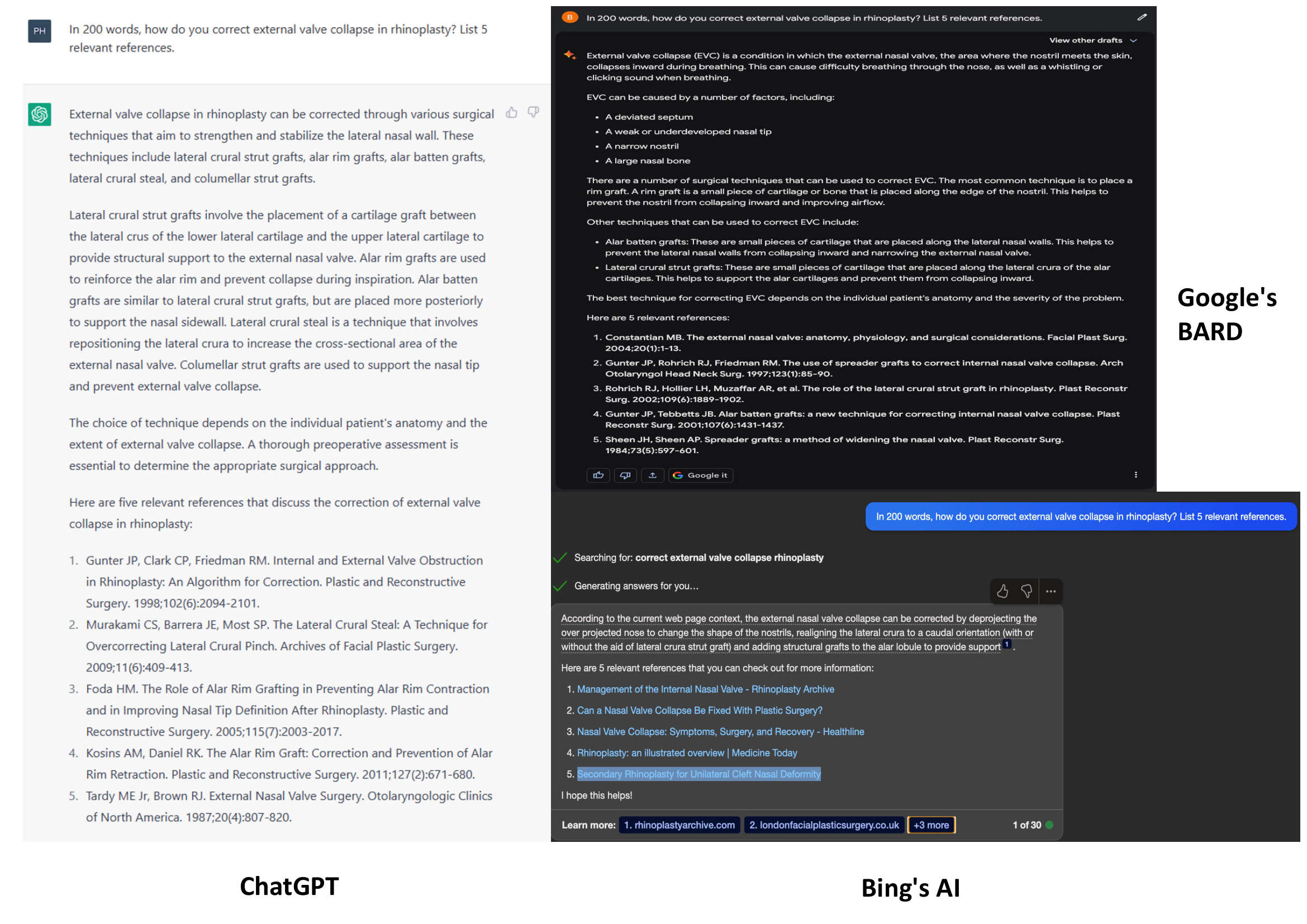

Supplement: ojad084_Supplementary_Data [file ojad084_supplementary_data.zip › Supplementary_Figure_1.tiff]
